# An aligned framework of actively collected and passively monitored clinical outcome assessments (COAs) for measure selection

**DOI:** 10.1038/s41746-024-01068-x

**Published:** 2024-03-16

**Authors:** Christine Manta Campbell, Courtney Webster, Megan Parisi, Roya Sherafat-Kazemzadeh, Jessica Braid, Thomas Switzer, Marcelo Alves Fávaro, Caprice Sassano, Andrea Coravos

**Affiliations:** 1 HumanFirst Inc., San Francisco, CA USA; 2 Nymbly LLC, Seattle, WA USA; 3 Syneos Health®, Morrisville, NC USA; 4 Mapi Research Trust, Lyon, France; 5 Roche Products Ltd., Welwyn Garden City, UK; 6 Genentech, Inc., South San Francisco, CA USA; 7 Harvard-MIT Center for Regulatory Science, Boston, MA USA

**Keywords:** Clinical trial design, Health services, Biomarkers

## Abstract

Regulators increasingly require clinical outcome assessment (COA) data for approval. COAs can be collected via questionnaires or digital health technologies (DHTs), yet no single resource provides a side-by-side comparison of tools that collect complementary or related COA measures. We propose how to align ontologies for actively collected and passively monitored COAs into a single framework to allow for rapid, evidence-based, and fit-for-purpose measure selection.

Selecting endpoints for a clinical study is complex and research-intensive. Determining the “right” endpoints—those that demonstrate safety, efficacy, and measure concepts that matter^1^ to patients—is required for regulatory approval and reimbursement. Patient outcomes can be classified as biomarkers^2^, which represent a physiological process, or as clinical outcome assessments (COAs)^3^, which reflect how a person feels, functions, or survives^2^. Traditionally, COAs are collected through “active” completion of questionnaires by patients (PROs), clinicians (ClinROs), or observers such as care partners (ObsROs); or through completion of a standardized task (a PerfO). Measures from sensor-based digital health technologies (DHTs) can be either biomarkers or COAs, as others have described^4^. When a DHT is reporting COA data, the data are considered “actively collected” (a PerfO) if the participant is performing a standardized task (e.g., the 6-minute-walk test or the endurance shuttle walk test), as the standardized task can lead the participant to “actively” influence or inform the data collected at the time. If the DHT is passively monitoring a participant’s real-world activity, it is considered a DHT-Passive Monitoring COA^5^ (Fig. 1).

**Fig. 1:**
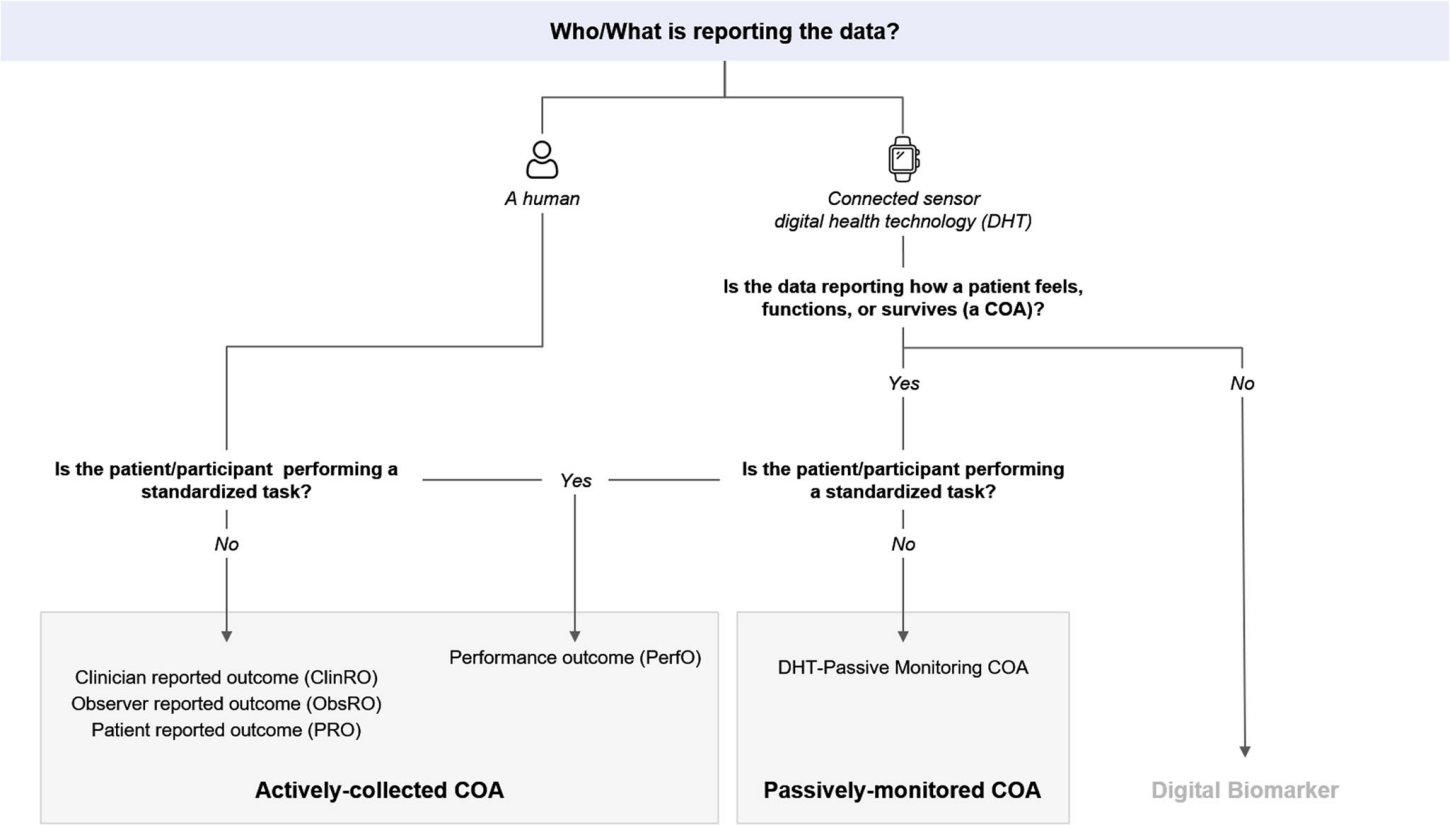
Patient outcomes are either a biomarker or a clinical outcome assessment (COA), and COAs can be collected passively or actively. Icons are by flaticon.com.

When selecting which COAs will represent study endpoints, researchers can identify a questionnaire, standardized task, DHT, or combination thereof that can potentially measure the same or related outcomes (Supplementary Fig. 1). Questionnaires and standardized tasks provide a point-in-time perception or measurement, whereas COAs collected by a DHT can deliver continuous, “real-world” information. The use of DHT-Passive Monitoring COAs is growing, though they are more commonly used to supplement rather than substitute the more established COAs. All of these data-collection options deliver value, but it is critical to determine how each is best suited to the research question at hand. Traditionally, researchers have had to review literature, published trial data, and various databases to determine which tool is well validated and fit-for-purpose to collect evidence within their context of use. This process is labor- and time-intensive.

Many databases use an ontology to organize, label, and categorize data elements so that users can understand and explore the data presented. Databases organizing COA data include Mapi Research Trust’s ePROVIDE™ (https://eprovide.mapi-trust.org/) and HumanFirst’s Atlas™ (https://www.gohumanfirst.com/atlas/platform), as well as open-access resources such as Northwestern University’s HealthMeasures (https://www.healthmeasures.net/index.php) and Digital.Health (https://digital.health/). We propose that there is enough alignment between existing ontologies to generate a single framework, allowing researchers to identify and then compare all actively collected versus passively monitored tools for the same or similar concepts.

When evaluating whether to use questionnaire(s), DHT(s) and/or standardized task(s), researchers ask: “Is the tool well validated in my target population?,” “Does the tool directly/indirectly measure a concept that is meaningful to patients?,” and “Can the tool be deployed (e.g., shipped, translated, operated, etc.) in my geographic regions of interest?” For drug development research, they question: “Are the data robust enough to support regulatory approval?” A unified framework would offer both utility and efficiency gains by addressing these questions in a single place. It would also enable exploration for broader queries such as: “Which questionnaire(s), DHT(s), and standardized tasks have been used to measure COAs relevant to atopic dermatitis?” or “How has scratch been measured actively and/or passively?”

To assess the feasibility of this aligned framework, we began by comparing the ontologies underlying actively collected COAs from ePROVIDE™ and passively monitored COAs from Atlas™ (Supplementary Fig. 2). We selected these proprietary databases because of their breadth and depth, but so long as a defined ontology is used, open-access resources can also be aligned. Therapeutic area, medical condition, and intended population can serve as anchor points to map COAs to one another. Other data elements, such as domain/concept of interest and objective/measures, could be aligned after subject-matter experts collaborate to develop a consensus on appropriate definitions. Data elements that are unique to an actively collected COA (e.g., translations, question types) or a passive-monitoring COA (e.g., API availability, sensor type) should be retained. These are important for practical considerations, such as accessibility or usability.

With the conceptual alignment defined, we then developed an actual proof-of-concept. For researchers designing a clinical study—for example, “A phase 3 study of drug XY1234 for atopic dermatitis (AD)”—this framework could help identify a PRO for the perception of itch (pruritus) and a sensor for the passive measurement of scratching (Fig. 2). These are cardinal, complementary symptoms of AD that matter both clinically and to the patient. This framework demonstrates that the Worst Itch Numerical Rating Scale (WI-NRS) questionnaire and actigraphy align for both the therapeutic indication (atopic dermatitis) and the concepts of interest (itch and scratch). The WI-NRS reports the participant’s assessment of itch severity over the past 24 h, whereas actigraphy measures scratch frequency, duration, and intensity through continuous passive monitoring.

**Fig. 2:**
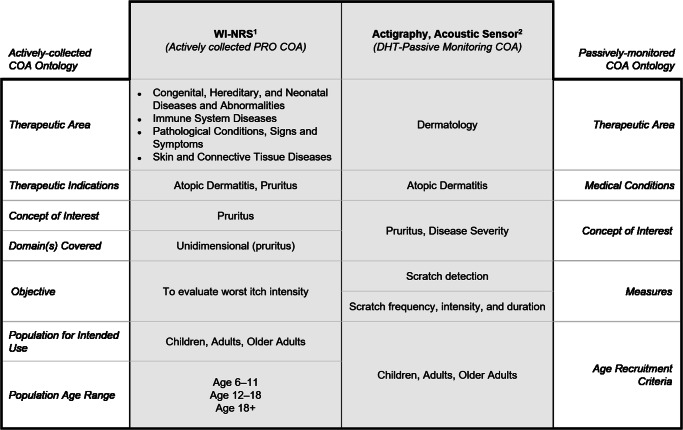
An excerpt of aligned data elements to compare the WI-NRS with actigraphy for the measurement of itch. ^1^Content available within MAPI Trust’s ePROVIDE™ platform (https://eprovide.mapi-trust.org/). ^2^Content available within HumanFirst’s Atlas™ platform (https://www.gohumanfirst.com/atlas/platform).

This comparison enables fit-for-purpose measure selection for this clinical study. For example, researchers may decide to use only actigraphy in a pediatric population, for whom a PRO might be difficult to administer. Or if used together, actigraphy could measure objective changes in the frequency, intensity, and duration of scratching and the PRO would serve to contextualize these changes in terms of a change of itch intensity. Researchers would expect to see directional reductions in both measures as a potential positive response to therapy.

As an additional example of the framework’s utility, we present another use case to compare the St. George’s Respiratory Questionnaire (SGRQ) with the Strados^TM^ Labs’ RESP Biosensor (Supplementary Fig. 3). The active and passive measurement of cough is relevant to a number of pulmonary conditions and has received renewed interest with the COVID-19 pandemic.

We then demonstrate how the framework could be used as part of developing a schedule of assessments for the study described above in Fig. 3. In this example, a researcher would begin by searching the framework for an indication of interest, narrowing to the meaningful aspect of health and concept of interest, and then “filtering” the data based on a measure of interest. The framework would display results based on these instructions and present a subset of data (such as number of publications and validation information) that would help the researcher select items to further compare. The researcher can “drill down” into more detailed information for each tool as well, and then return to the search/compare view. Comparison and drill-down can be iterated to help the researcher select fit-for-purpose tools for evidence collection. The researcher could use the information generated by the framework along with the frequency and cadence of each COA, the defined dosing schedule, and other study-specific criteria outside of the framework to develop a schedule of assessments for their clinical study.

**Fig. 3:**
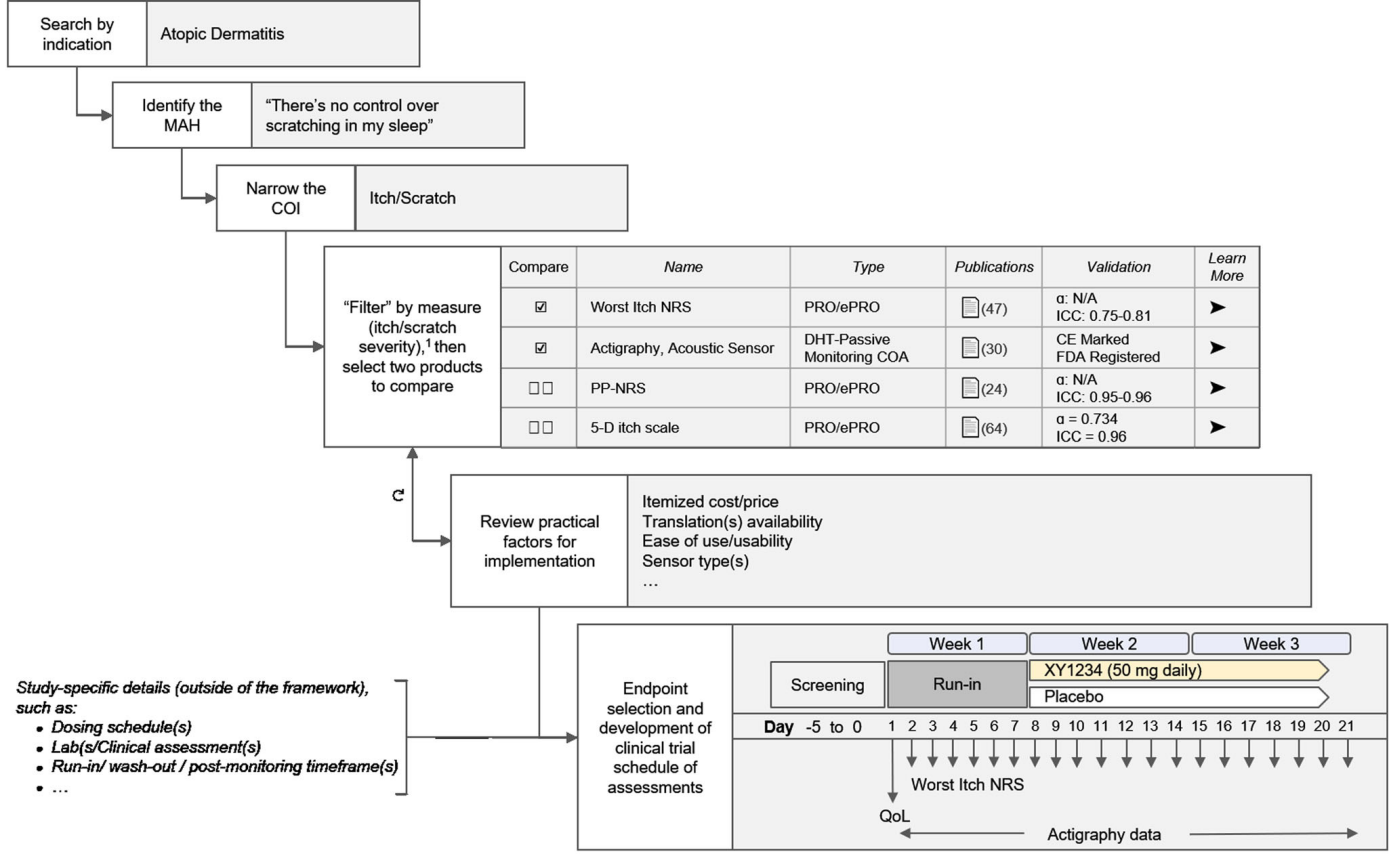
Using the aligned framework to identify and compare measurement tools to develop a clinical protocol schedule of assessments. ^1^Content available within HumanFirst’s Atlas™ platform (https://www.gohumanfirst.com/atlas/platform).

We emphasize the importance of subject-matter expert collaboration to align more complex data elements within this framework. This allows the framework to display complementary elements, such as the sensation of itch compared with the physical action of scratch in Fig. 2, as well as identical elements, such as comparing cough with cough as shown in Supplementary Fig. 1.

Decisions to select fit-for-purpose tools for measures have been made for many years now, but through laborious research and comparison. Once those decisions are made, the evidence may or may not become available publicly—leading the next researcher to repeat the burdensome process again. The effort and difficulty of the current process results in many researchers struggling to select endpoints, mimicking prior studies’ endpoints despite not representing real-world data, a general lack of focus on relevant endpoints and the inability to correlate or supplement actively collected data with passively collected data. The long-term value of this framework is its living, dynamically updated repository of evidence to encourage the collection of both actively-reported and passively-collected data. For example, both Atlas^TM^ and ePROVIDE^TM^ are maintained through automated and semi-automated mechanisms with reviews for quality and accuracy. These techniques also drive the aligned framework to maintain up-to-date information in addition to historical findings.

While this framework overcomes many current issues, decisions will still need to be made by trained researchers. The quality of the evidence, the similarity of the populations of interest, and ensuring the COA’s validation is sufficient for the intended purposes are critical factors to both actively collected and passive-monitoring COAs.

The power of this foundational framework lies in its ability to provide illustrative, side-by-side comparisons of actively collected and passive-monitoring COAs, thus enabling rapid, evidence-based selection. Although not yet comprehensive, this shared language will enable continued advancement in technologies and tools. Future work will address expanded use cases as well as more advanced or complex scenarios, such as composite COAs.

This framework enables more rapid measure selection, which would decrease study startup timelines, and could allow researchers to reduce the number of assessments through more efficient endpoint selection. From 2015 to 2021, endpoints in individual trials increased by 37% with an average of 25.8 endpoints per trial^6^. Reducing the number of assessments per protocol would reduce the burden on participants, which would lower barriers to entry and promote continued engagement. This could increase response rates and reduce issues with missing data, as well as reduce downstream effort and cost by investigators and research sites. Ultimately, demonstrating therapeutic efficacy more efficiently will benefit patients awaiting new ways to better manage or treat disease.

This aligned, dynamically maintained framework is a shared language to support the industry’s advancements in modern trial design, such as:

- Supporting regulators in assessing and accepting sensor-based endpoints,
- Developing new ways to validate sensor-based measurements and eliminate bias,
- Using this large, organized dataset to train language models and/or generative AI,
- Aligning meaningful-change thresholds to evaluate how the clinical interpretation of a score on an actively collected COA compares with a DHT Passive-Monitoring COA,
- Increasing flexibility in substituting actively collected COAs with DHT Passive-Monitoring COAs,
- Minimizing rater variability by substituting clinician-reported outcomes (ClinROs) with DHT Passive-Monitoring COAs,
- Substituting standardized tasks with DHT-Passive Monitoring COAs^7^, or
- Developing new ways to validate actively collected COA effectiveness across various populations, which could lead to more easily adapted modalities and languages to match the modern pace of language change.

We believe this framework would encourage the collection of both actively reported and passively collected data, allowing us to better understand disease burden and promote patient-centric drug development. The ultimate objectives are to minimize protocol complexity and participant burden, accelerate clinical studies, and drive innovation in clinical studies to support drug discovery and approval.

## Methods

### Terminology definitions

Terminology associated with developing digital technology is currently evolving and can be highly nuanced. We began by ensuring that the following terms were accurate and clarified regarding the context of use within this work. A summary of terminology clarifications is provided herein, and Fig. 1 was developed to provide a concise clarification of terms based on reporter, the intent for how the collected data may be used, and the context of the data collection.

DHT is a broad term that can refer to different uses of digital technology in clinical research and patient care, such as telehealth, medical devices, etc. For the purposes of this paper, we used a narrow definition of DHT that refers to use of sensors in products such as wearables or portable monitors to capture certain outcomes.

The US Food and Drug Administration (FDA) has not defined how to distinguish a PerfO from a DHT Passive-Monitoring COA when a DHT is reporting COA data. We propose that the activity performed by the participant during the measurement classifies it as actively collected (a PerfO) or passively monitored (a DHT Passive-Monitoring COA), as described herein. This definition aligns with how the FDA assigned “COA type” in its Clinical Outcome Assessments (COA) Qualification Program Submissions^5^.

### Comparison of ontologies and framework development

To generate Supplementary Fig. 2 and assess feasibility, we compared the ontologies comprising the MAPI Research Trust’s ePROVIDE™ database and the HumanFirst’s Atlas™ database. Subject matter experts compared the data elements comprised within and aligned which reported the same or similar content, whether the information was actively collected or generated through passive monitoring. We acknowledged that to present an accurate and comprehensive alignment of these ontologies, the assessments within each therapeutic area/condition would require a collaborative evaluation with clinical and technical expertise. For example, we identified that the data element “domain” from the ePROVIDE™ ontology and the data element “concept of interest” from the Atlas™ ontology could be broad (e.g., “symptoms” or “quality of life”) or could include clinical terminology (e.g., pruritus). The data element “objective” in the ePROVIDE™ ontology and the data element “measures” in the Atlas™ ontology must be carefully evaluated so that side-by-side and complementary comparisons can be searched and accurately presented within the proposed aligned framework. We have provided an example of a side-by-side comparison of “cough” to “cough” in Supplementary Fig. 2 and an example of a complementary comparison of “itch” to “scratch” in Fig. 2.

We selected the actively collected COA questionnaires and the DHT Passive-Monitoring COAs for Fig. 2 and Supplementary Fig. 2 after evaluating their breadth of use (e.g., are these examples widely recognized by the applicable scientific community and do they have robust evidence bases?). We also evaluated which exemplary comparisons would emphasize the utility of the proposed framework and would represent a unique contribution as well. We acknowledge that these examples offer simpler comparisons, with complex advanced or complex scenarios (such as composite COAs) reserved for future work.

Finally, we evaluated how the information presented by the proposed framework would integrate into existing workflows in drug development. There is tremendous scientific value in consolidating the information proposed within, but our priority was that this information deliver measurable improvements to patients in urgent need of more efficient and affordable treatments.

### Reporting summary

Further information on research design is available in the Nature Research Reporting Summary linked to this article.

### Supplementary information

Supplemental Material

Reporting Summary

## Data Availability

The full ontology that comprises MAPI Research Trust’s ePROVIDE™ database is not available due to its proprietary nature, though partial data are available for free. The ontology that comprises HumanFirst’s Atlas™ database is also not available due to its proprietary nature, and similarly, partial data are available for free. These data were used to create Fig. 2 and Supplementary Fig. 3. Data associated with the WI-NRS in Fig. 2, the WI-NRS, the PP-NRS, and 5-D itch scale in Fig. 3, and the St. George’s Respiratory Questionnaire (SGRQ) in Supplementary Fig. 3 are available at MAPI Research Trust’s ePROVIDE™ (https://eprovide.mapi-trust.org). Terms and conditions apply. For further information on availability and terms of access, please submit a request on the platform. The full dataset associated with actigraphy in Fig. 2 and the Strados^TM^ Labs’ RESP Biosensor in Supplementary Fig. 3 is available for a fee through a subscription to HumanFirst’s Atlas™ (https://www.gohumanfirst.com/atlas/platform). Terms and conditions apply.

## References

[1] Manta C, Patrick-Lake B, Goldsack JC (2020). Digital measures that matter to patients: a framework to guide the selection and development of digital measures of health. Digit. Biomark..

[2] FDA-NIH Biomarker Working Group. *BEST (Biomarkers, EndpointS, and other Tools) Resource* (National Institutes of Health, 2016).

[3] Coravos A (2019). Digital medicine: a primer on measurement. Digit. Biomark..

[4] Izmailova, E. S., Guo, C. & Coons, S. J. Conflicting terminology in digital health space: a call for consensus. *Appl. Clin. Trials* **32**, 9–10 (2023).

[5] U.S. Food and Drug Administration. Clinical outcome assessments (COA) qualification program submissions. https://www.fda.gov/drugs/clinical-outcome-assessment-coa-qualification-program/clinical-outcome-assessments-coa-qualification-program-submissions (2021).

[6] Tufts Center for the Study of Drug Development. Protocol design scope and execution burden continue to rise, most notably in Phase III. *Impact Rep*. **25**, 1–4 (2023).

[7] European Medicines Agency. Draft qualification opinion for stride velocity 95th centile as primary endpoint in studies in ambulatory Duchenne muscular dystrophy studies. https://www.ema.europa.eu/en/documents/regulatory-procedural-guideline/draft-qualification-opinion-stride-velocity-95th-centile-primary-endpoint-studies-ambulatory_en.pdf (2023).

