# Supplementary material for: An aligned framework of actively collected and passively monitored clinical outcome assessments (COAs) for measure selection
Source: NPJ Digit Med. 2024 Mar 16;7:71. doi: 10.1038/s41746-024-01068-x (PMC10944461; doi:10.1038/s41746-024-01068-x)
Supplement: Supplementary file 1 — Supplemental Material [file 41746_2024_1068_MOESM1_ESM.pdf]

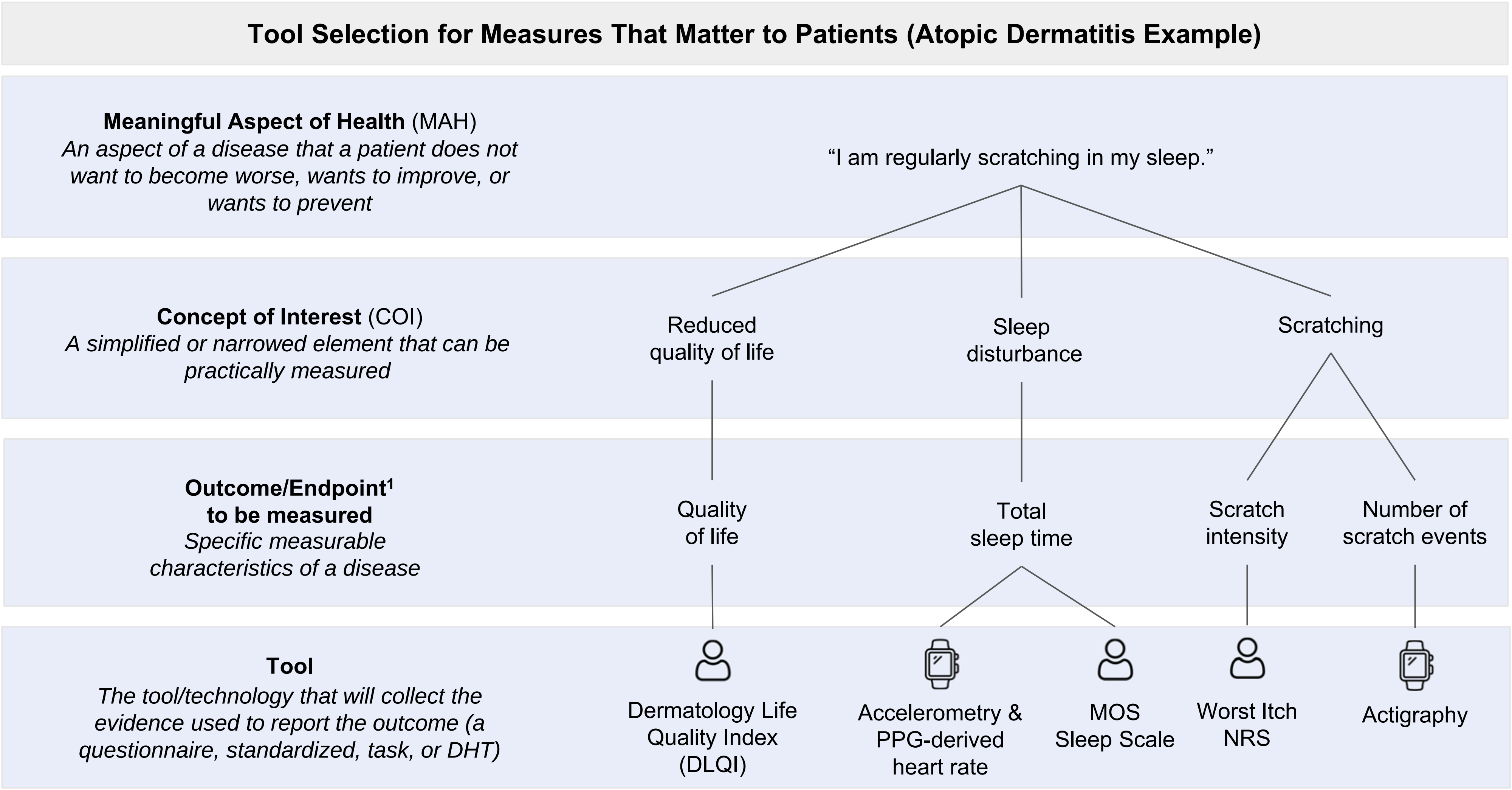

Supplementary Figure 1: Identifying, comparing, and then selecting which tools will collect evidence to support regulatory submission.

[1] If conducting research (e.g., a clinical trial), the research team will also define an endpoint to be measured, such as 'Average change in time to sleep onset 1 month from baseline'. Content derived from Manta C et al. Digit Biomark 2020;4 (3):69–77 and More than Skin Deep "Voice of the Patient Report" (2020) http://www.morethanskindeep-eczema.org/report.html, HumanFirst team analysis.

Actively Collected COA Ontology	Alignment Type	Passive Monitoring COA Ontology
Basic Description	*Aligned*	Description
Therapeutic Area	*Aligned*	Therapeutic Area
Therapeutic Indications [1]	*Aligned*	Medical Conditions [4]
Concept of Interest [2]<br>Domain(s) Covered [2]	*Aligned*	Concept of Interest
Objective	*Aligned*	Measures
Population for Intended Use<br>Population Age Range	*Aligned*	Age Recruitment Criteria [5]
References	*Aligned*	Evidence
Authors<br>Copyright<br>Type of COA[3]<br>Original Language(s)<br>Translations<br>Number of Questions<br>Types of Questions<br>Administration Mode<br>Data Collection Mode<br>Time for Completion<br>Scoring Methodology<br>Recall/Observation Period<br>…	*Unique*	FDA Classification<br>CE Mark<br>Sensor Type<br>Wear Location[6]<br>Form Factor<br>Software<br>API Availability<br>SDK Availability<br>Access to Preprocessed Data<br>Privacy Policy<br>Security Policy<br>Collection Interval [7]<br>…

Supplementary Figure 2: Mapping the conceptual overlap between actively-collected and passive monitoring COA ontologies

[1] The MAPI ePROVIDE™ platform classifies therapeutic indications based on MeSH

[2] For COAs, the authors have combined Domain and Concepts of Interests as the existing ontologies use different terminology.

[3] This refers to who or what reports the data (e.g., PRO, ClinRO, ObsRO, PerfO)

[4] The HumanFirst Atlas™ platform classified medical conditions based on ICD10

[5] The HumanFirst Atlas™ platform gathers and organizes this data from reported evidence

[6] The location of a sensor, if worn (e.g. wrist, chest, ankle) or if a sensor is contactless (e.g., sensors that use infrared cameras or motion sensors rather than being in direct contact)

[7] Continuous versus intermittent

Actively collected COA Ontology	**St. George's Respiratory Questionnaire (SGRQ)**[1] *(Actively collected PRO)*	**Strados™ Labs' RESP Biosensor**[2] *(DHT-Passive Monitoring COA)*	*Passively monitored COA Ontology*
Therapeutic Area	Respiratory Tract Diseases	Pulmonology/Respiratory	*Therapeutic Area*
Therapeutic Indications	Asthma, COPD, Bronchiectasis,	Asthma, COPD, Pulmonary Fibrosis	*Medical Conditions*
Concept of Interest	• Respiratory Symptoms	Respiratory Sounds	*Concept of Interest*
Domain(s) Covered	• Symptoms<br>• Activity [4]<br>• Impact on daily life [4]		
Objective	Health-related quality of life	Cough	*Measures*
		Wheezing	
Population for Intended Use	Adult	Children[3], Adolescents[3], Adult, Older Adult	*Age Recruitment Criteria*
Population Age Range	18 years old and over		

Supplementary Figure 3. An excerpt of aligned data elements to compare the St. George's Respiratory Questionnaire (SGRQ) to the the Strados™ Labs' RESP Biosensor for the measurement of cough.

[1] Content available within MAPI Trust's ePROVIDE™ platform (https://eprovide.mapi-trust.org/)
[2] Content available within HumanFirst's Atlas™ platform (https://www.gohumanfirst.com/atlas/platform)
[3] FDA-approved for adult use, though a clinical trial is ongoing to support use in pediatric population (https://classic.clinicaltrials.gov/ct2/show/NCT06112080)
[4] These domains are included within the SGRQ, though the most direct comparison will be the Symptom domain compared with the Respiratory Sounds concept of interest.
